# Portable Nanoparticle-Based Sensors for Food Safety Assessment

**DOI:** 10.3390/s151229826

**Published:** 2015-12-05

**Authors:** Gonca Bülbül, Akhtar Hayat, Silvana Andreescu

**Affiliations:** 1 Department of Chemistry and Biomolecular Science, Clarkson University, Potsdam, NY 13699-5810, USA; bulbulg@clarkson.edu; 2 Interdisciplinary Research Centre in Biomedical Materials, COMSAT Institute of Information Technology (CIIT), Defence Road, Off Raiwind Road, Lahore 54000, Pakistan; akhtarhayat@ciitlahore.edu.pk

**Keywords:** nanoparticle, detection, portable, sensor, food contamination, toxicity

## Abstract

The use of nanotechnology-derived products in the development of sensors and analytical measurement methodologies has increased significantly over the past decade. Nano-based sensing approaches include the use of nanoparticles (NPs) and nanostructures to enhance sensitivity and selectivity, design new detection schemes, improve sample preparation and increase portability. This review summarizes recent advancements in the design and development of NP-based sensors for assessing food safety. The most common types of NPs used to fabricate sensors for detection of food contaminants are discussed. Selected examples of NP-based detection schemes with colorimetric and electrochemical detection are provided with focus on sensors for the detection of chemical and biological contaminants including pesticides, heavy metals, bacterial pathogens and natural toxins. Current trends in the development of low-cost portable NP-based technology for rapid assessment of food safety as well as challenges for practical implementation and future research directions are discussed.

## 1. Introduction

Food safety remains a major concern worldwide. The presence of unsafe levels of chemical and biological toxins in food represents a serious threat to the safety of the food supply and public health. According to the World Health Organization (WHO), foodborne illnesses predominantly affect the economy of underdeveloped nations. Food safety issues in developing countries are widely recognized; estimates indicate around 1500 annually diarrheal episodes occurring globally, 75% of which are attributed to biological contamination of food, resulting in ~3 million deaths [1]. The WHO has placed food safety among its top 11 priorities. The U.S. Centers for Disease Control and Prevention have estimated that 48 million Americans get sick because of contaminated food, 128,000 are hospitalized and 3000 die due to foodborne diseases [2].

In order to manage and overcome the problems related to foodborne illnesses, it is important to develop easy-to-use tests that can rapidly measure the presence of toxic contaminants in food so that remedial actions can be taken. Most analysis of chemical and biological contaminants is performed in centralized laboratories and only a limited number of samples can be tested. More effective methods are needed to facilitate the screening of potentially contaminated samples remotely at food production and handling locations where resources or specialized equipment are not available. Several types of enzyme-based and bioaffinity assays have been reported as alternatives to conventional analytical instrumentation [3,4,5,6,7], albeit with few examples of food safety applications [8,9,10,11]. While progress has been made, these assays are still complex and costly, involve multiple analysis steps, addition of sensitive reagents and expensive instrumentation; and are not portable. Easy-to-use inexpensive methods that could be deployed remotely to site locations would significantly improve management and control of food quality and safety.

Nanotechnology-derived products have provided a wide range of material candidates that can be used to increase portability and enhance stability, selectivity and sensitivity of sensors and analytical measurement technologies. Nanotechnology is most widely used in electronics, sensing, biomaterials and catalysis [12,13,14] and more recently has made its way into the food industry [15]. Current applications comprise: nanomaterial-based encapsulation and delivery systems, antibacterial nanoparticles, NP additives for increasing the flavour and shelf-life of food products and for, tracking, tracing and brand protection [15,16]. Developments in the control of size, surface properties and assembly of NP systems provide opportunities for the development of advanced sensing systems and portable instrumentation that incorporate nanotechnology enabled solutions. Colorimetric [17] and electrochemical [18] detection systems have already been integrated with low-cost platforms such as patterned paper enabling on-site analysis. These portable, low cost and user-friendly sensors have been developed as alternative to conventional analytical methods for point of care medical diagnosis [19], environmental monitoring and food quality control [20]. Application of screen printed carbon electrodes (SPCE) as a portable platform in electrochemical sensors for environmental monitoring and food quality control have been extensively reported [21]. This review summarizes recent advancements in the design and development of NP-based sensors for assessing food safety. Selected examples the from literature on NP-based detection schemes, operational parameters and applications for measurement of food contaminants, as well as challenges for practical implementation and future research directions are discussed.

## 2. Common Types of Nanostructures in Nanotechnology-Based Sensing Approaches

### 2.1. Gold NPs

**Figure 1:**
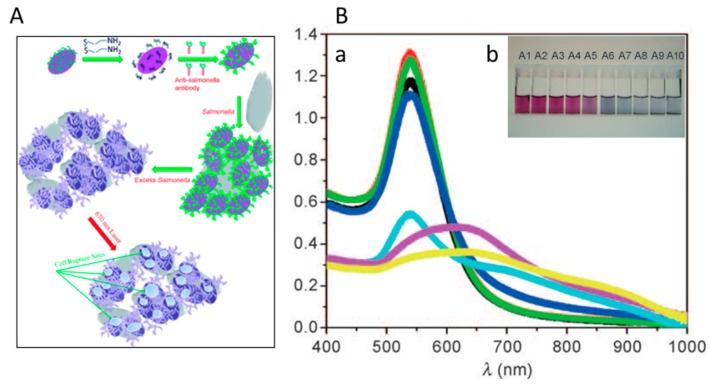
(**A**) Schematic of a colorimetric AuNPs based approach to selectively target and destroy pathogenic bacteria with antibody-conjugated oval-shaped AuNPs; (**B**-**a**) Absorption-profile of anti-salmonella-antibody-conjugated oval-shaped AuNPs in the absence of bacteria (**red**) due to the addition of 10^6^ CFU/mL *E. coli* bacteria (**green**) or different concentrations of *Salmonella bacteria* (10 (**black**), 50 (**dark blue**), 10^3^ (**light blue**), 10^4^ (**pink**), and 10^5^ bacteria (**yellow**)). The new band at around 680 nm following the addition of *Salmonella*, indicates the aggregation of the AuNPs (**B**-**b**) Photograph shows colorimetric change upon the addition of (A1) 10, (A2) 50, (A3) 100, (A4) 500, (A5) 1000, (A6) 5000, (A7) 10,000, (A8) 50,000, (A9) 100,000, and (A10) 500,000 *Salmonella* (reprinted with permission from [41]).

Most common nanotechnology-based sensing approaches utilize noble metal NPs such as gold [22] and silver [23,24,25,26]. Such applications are enabled by the useful optical properties of these NPs which can be tuned by changing the size, shape, local environment, and the synthesis method [27,28]. AuNPs have been used as carriers [29] for the biorecognition element such as antibodies or aptamers and as labels for signal transduction and amplification [30]. The basis for the absorption-based colorimetric sensing involves aggregation-induced interparticle surface plasmon coupling of AuNPs which results in a visible color change from red to blue. This concept has provided a practical platform for detection of any target analyte that triggers the AuNPs aggregation or re-dispersion [31]. AuNPs have been widely used to increase surface area and conductivity in electrochemical sensors. A variety of colorimetric and electrochemical assays based on AuNPs have been reported for the detection of chemical contaminants such as alkali and alkaline earth metal ions [32,33,34], heavy metal ions [35,36,37,38] and for assessment of microbiological food contamination like bacteria [39,40]. An example of colorimetric AuNP-based sensing for detection of pathogenic bacteria is shown in Figure 1. A detailed review on AuNP-based sensing has been published [31].

### 2.2. Silver NPs

Several sensing systems based on the optical properties of silver NPs (AgNPs) have been reported [24,42]. The color change between dispersed and aggregated AgNPs from yellow to brown can be associated with the concentration change of a target molecule [43]. Based on this principle, various AgNP-based assays have been developed for detection of metal ions [44], proteins [45], melamine [46] and pesticides [47].

**Figure 2:**
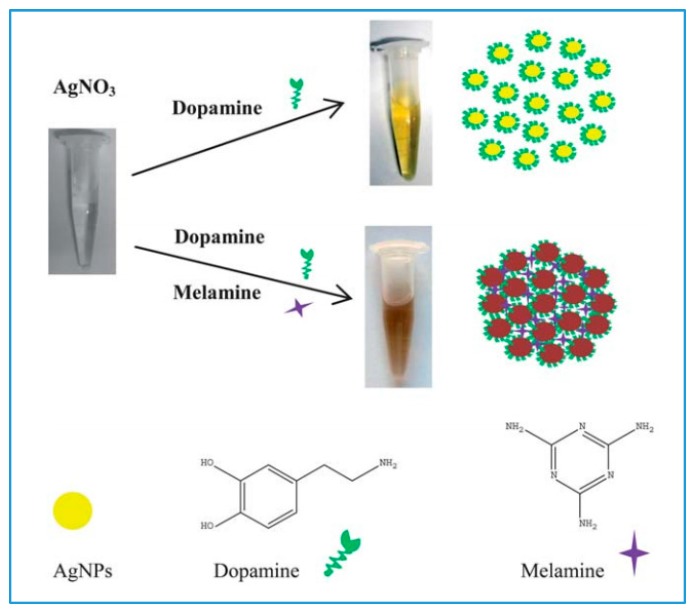
Schematic illustration of colorimetric method with AgNPs/dopamine system for the detection of melamine (reproduced from [48] with permission of The Royal Society of Chemistry).

Figure 2 shows an example of sensing of melamine based on the optical properties of AgNPs [48]. As compared to AuNPs, AgNPs retain higher extinction coefficients and have lower cost [49]. However, less focus has been placed on AgNPs based sensing due to the following limitations: (1) the functionalization of AgNPs can cause chemical degradation of NPs to silver ions and (2) the surface of AgNPs can be easily oxidized [26].

### 2.3. Cerium Oxide NPs

**Figure 3:**
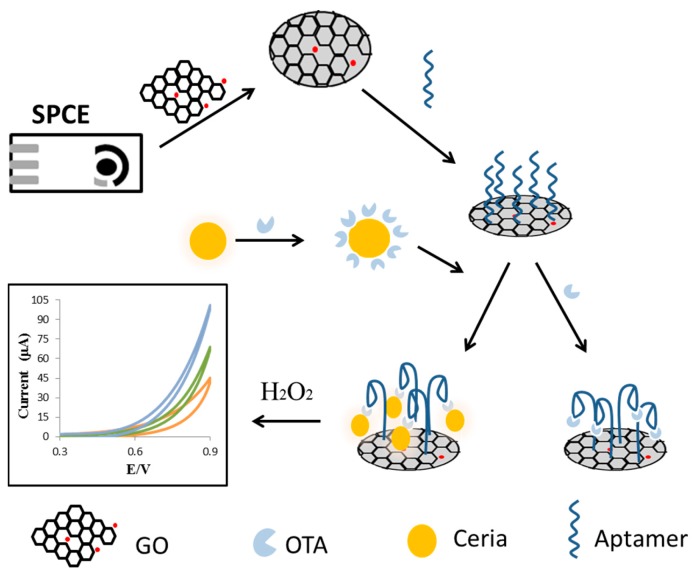
Non-enzymatic electrochemical aptasensor based on the use of a nanoceria tag and graphene oxide (GO) on a screen-printed electrode (SPCE) for the detection of ochratoxin A (OTA) (reproduced from [50] with permission of The Royal Society of Chemistry).

The use of cerium oxide NPs, or nanoceria as an active sensing component in portable assays is rapidly emerging [50,51,52,53]. Nanoceria has the ability to change redox states and surface properties due to the presence of dual reversible oxidation state of cerium Ce(III)/Ce(IV) on the NP surface. Nanoceria particles have been found to possess peroxidase-, superoxide- and oxidase-like activity [54] suggesting that they could potentially replace these enzymes in the development of analytical assays [51]. Nanoceria is optically active and can develop unique color patterns depending upon specific interactions at their surface. These changes can be associated with a target analyte which make these particles an attractive choice from colorimetric sensing [55]. We have used the optical changes of nanoceria upon interaction with phenolics and H_2_O_2_ to fabricate portable colorimetric sensors for the detection of food antioxidants and glucose in which nanoceria acts as a colorimetric probe [51,55,56]. When used in conjunction with other metal oxides it is possible to establish a multi-array sensory panel in which each sensor provides a unique signature that could be used for cross-validation and increased accuracy [55,57]. Recently, we have utilized the enzyme mimetic properties of nanoceria to develop a portable enzyme-less electrochemical aptasensor for detection of food contaminants such as mycotoxins [50]. The general sensing design is shown in Figure 3.

### 2.4. Carbon Nanotubes and Graphene

Graphene is an isolated single atomic layer of graphite which is currently utilized in electrochemical analysis due to its exceptional conductivity. Since the investigation of exfoliation and characterization of graphene by Geim and Novoselov in 2004 [58], the remarkable electronic transport properties of individual graphene sheets have been demonstrated in many studies [59,60]. The large surface area, high conductivity and ease of modification with biomolecules have been applied to a variety of biosensing systems [61,62,63], particularly those with electrochemical detection [64,65]. Graphene-based composite materials have been fabricated for electrochemical detection of food contaminants such as bisphenol A [66,67], hydrazine and nitrite [68], organophosphorus pesticides [69] and bacteria [70]. In addition, colorimetric detection of melamine [71], ochratoxin A [72], and mercury (II) (Hg^2+^) and silver (I) (Ag^+^) [73] ions using graphene-based composite materials has also been reported. Applications of graphene-based nanomaterials have been reviewed [74].

### 2.5. Magnetic Nanoparticles

Magnetic nanoparticles (MNPs) have been mostly utilized as immobilization supports in sensing assays and in the development of immunomagnetic separations and magnetically loaded and controlled sensoring platforms [75]. Functionalized MNPs with a variety of surface groups are readily available, permitting development of different strategies for detection of a variety of analytes [76]. Their large surface area and the increased possibilities for enhancing the assay kinetics, control the loading and improve the immobilization efficiency are some of the advantages of MNPs, which make them one of the most widely used NPs for detection and removal of food contaminants [77]. A multiplexed magnetic microsphere immunoassay for detection of food pathogens developed by Kim *et al.* showed good operational performance in spiked foodstuff such as apple juice, green pepper, tomato, ground beef, alfalfa sprouts, milk, lettuce, spinach, and chicken washes [78]. Magnetic beads conjugated with bacteriophage were utilized for the detection of *E. coli* in drinking water with a detection limit of 10 cfu/mL after pre-enrichment [79]. A sensor design by integrating magnetic nanobeads for detection of organophosphate insecticides using acetylcholinesterase was demonstrated on screen printed carbon electrode surface [80].

### 2.6. Low-Cost Platforms for Portable NP-Based Detection

Two types of transducer platforms are preferred for the development of inexpensive portable NP-based sensors: (1) screen printed electrodes and (2) paper. The screen printing technology is a low cost process that has been extensively used in artistic applications and for design electronic circuits. In the 80s, the screen printing technology was extended to the fabrication of portable electrochemical sensors, making them more suitable for commercialization [81]. Biosensors based on screen printed electrodes offer the advantages of reduced cost, ease in automation and good reproducibility and sensitivity characteristics. Various nanomaterials including but not limited to carbon nanomaterials, CeO_2_, Au, Ag and ZnO NPs have been added as active sensing components to working electrodes to increase surface area, add catalytic properties and amplify electrochemical signals. Some approaches involve the addition of NPs or nanotubes in the composition of screen-printed inks [82]. In other procedures, nanomaterials are drop-casted in DMF/water or electrodeposited on the working electrode surface [83]. The use of nanomaterials in the design of SPCE electrodes provides the following benefits [76]: (a) immobilization support for biomolecules increasing stability and bioactivity; (b) mediator to promote electron transfer reactions, lower the working potential and prevent interferences problems, improving sensitivity and selectivity; (c) electroactive label for electrochemical striping techniques to generate an electrochemical signal; (d) catalyst to amplify the electrochemical signal, enhancing sensitivity. Several examples of disposable nanomaterials-based SPCE electrochemical biosensors have been reported for the detection of food contaminants including pesticides, bacterial toxins and mycotoxins as well as for the detection of food antioxidants [84]. Specific examples will be discussed in the following section.

**Figure 4:**
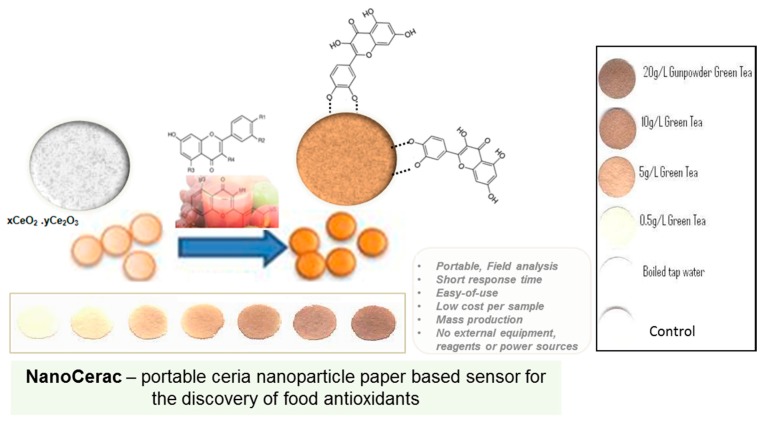
Operational concept of the NanoCerac assay based on surface-immobilized nanoceria particles for field analysis of antioxidants (reproduced from [55] with permission of The Royal Society of Chemistry).

Another type of material that has received significant attention as a sensing platform in the last few years is paper. Starting with Whitesides’ report in 2007, there has been a tremendous effort to develop paper-based low-cost sensors as alternatives to conventional methods for field analysis [17,85]. Paper is the simplest, most affordable and abundant material. Examples of paper bioassays include patterned paper fabricated by photolithography for detection of glucose and bovine serum albumin [17], inkjet-printed paperfluidic immuno-chemical sensing device [86], aptamer—NP-based lateral flow devices for detection of DNA sequences [87], inkjet-printed enzyme sensors for the detection of bisphenol A in field samples [88,89]. Paper based sensors are miniaturized, disposable and can be used for on-site analysis. Conductive materials can be added to modify the paper surface and enable electrochemical detection. These platforms have been integrated with colorimetric [90] and electrochemical [85,91] detection methods. The use of paper-based electrochemical sensors has been demonstrated for detection of analytes of interest in environmental monitoring, health care and food quality control [92]. Baxter *et al*. have proposed a simple and economical process to fabricate gold electrodes on paper using a camera flach sintering step [93]. Nie *et al.* [94] have integrated an electrochemical paper sensor with a commercial glucometer and have demonstrated applicability for on-site analysis of ethanol in food. The detection involved the enzymatic conversion of ethanol with alcohol dehydrogenase in the presence of β-NAD+. Ferricyanide was used as a mediator to enhance electron transfer [94]. Most paper based biosensors are still in their early developmental stage, especially in the field of food quality control. We have recently developed a portable and reagentless NP-based paper platform to detect oxidase enzyme substrates (e.g., glucose) [51] and polyphenols in food samples (e.g., wild mushrooms, wine, juice and green tea) [55] using the redox and surface chelating properties of nanoceria. Detection of the analyte was performed by quantifying the color change of the particles after reaction with the product of the enzymatic reaction (H_2_O_2_) or the polyphenol. All reagents needed for detection were immobilized onto paper. There was no need for addition of external reagents or the use of a power supply to perform analysis; the only step needed for analysis was the addition of the analyte. The enzyme sensor was very robust and stable over several months. The nanoceria-based antioxidant assay operates similar to a small sensor patch that changes color after contact with antioxidants (Figure 4). The sensor provided an optical signature of the antioxidant power of the sample [57]. When used in conjunction with other metal oxides it is possible to establish a multisensor panel in which each sensor provides a unique signature that could be used for cross-validation and increased accuracy [55,57]. This sensor has demonstrated applicability and excellent performances of field analysis of the brewing conditions for a large number of green tea samples [95].

## 3. NP-Based Technologies for the Detection of Biological and Chemical Contaminants

### 3.1. Current Status of Foodborne-Related Illnesses

The food industry is the largest manufacturing sector in the world [96]. Unwashed foods, inadequate processing or cooking, poor quality of water can be a direct source of foodborne diseases. Excessive use of agrochemicals (pesticides, plant growth regulators, veterinary drugs) and presence of environmental contaminants and harmful microorganisms such as pathogenic bacteria, viruses, or parasites are the common causes of food contamination. According to the U.S. Food and Drug Administration (US-FDA) the primary contaminants leading to foodborne illness are pathogenic microorganisms including: *Bacillus cereus*, *Clostridium botulinum*, *Escherichia coli* (*E. coli*)*,* hepatitis A*, Listeria monocytogenes*, *Noroviruses*, *Salmonella* and *Staphylococus aureus,* among others. Other contaminants, such as the toxic fungal metabolites known as mycotoxins are rapidly emerging. Currently, there are limited methods for field detection of toxins and foodborne pathogens, making early identification of a possible contamination difficult. To prevent and manage contamination, there is a need to develop portable and inexpensive detection tools that can provide effective screening and facilitate the analysis at food production and handling locations where specialized equipment is not available. Being able to assess the safety of food from the production process until it reaches the consumer is important for assessing food related health risks. The integration of NPs in the analysis methods for detection of food contaminants has improved the detection sensitivity and increased portability. However, the implementation of these methods for the analysis of real samples still remains as a challenge due to the uncontrollable properties of NPs in complex environments such as aggregation, non-specific signals due to interferences and chemical reactions with food constituents. In the following sections we discuss recently reported NP-based portable assays for the detection of chemical and biological contamination with their analytical performance characteristics for the analysis of food.

### 3.2. Detection of Microbial Contamination

The presence of pathogens in food or water can cause foodborne infections. They comprise bacteria, viruses, fungi and parasites [97]. The most common foodborne infections are caused by *Campylobacter* spp., *Salmonella* spp. and *E. coli* O157:H7 [98,99]. The conventional method for food pathogen detection is colony counting (CFU) on an agar plate which takes 2–3 days for initial results, and up to 1 week for confirming pathogen specificity [98]. This method is time consuming and laborious. Polymerase chain reaction (PCR) and enzyme-linked immunosorbent assay-based (ELISA) can be used as alternative to traditional CFU methods [100]. While these methods have high sensitivity and selectivity, both PCR and ELISA are still slow, labour-intensive and costly to implement in resource-limited settings. Current research activities target development of methods that can be adapted on portable platforms to enable rapid testing of a range of pathogens with potential for on-site analysis [101]. This section provides examples of recently developed NP-based assays for the detection of pathogens, with specific examples for *E. coli* and *Salmonella* spp.

**Figure 5:**
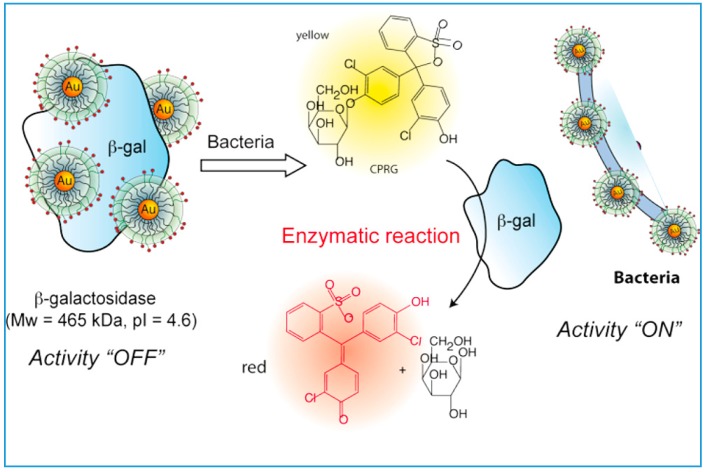
The schematic representation of the sensing mechanism of the colorimetric enzymatic nanocomposite biosensor for the detection of *E. coli* (reprinted with permission from [40]; copyright 2015 American Chemical Society).

*E. coli* O157:H7 [102], which can contaminate ground beef, raw milk, poultry products, cold sandwiches, vegetables, and drinking water supplies [103,104,105], is recognized as one of the most dangerous pathogens. Most methods designed for on-site detection of *E. coli* involve competitive displacement assays [39] or immuno-chromatographic test strips [106]. Miranda *et al.* developed a hybrid colorimetric enzymatic nanocomposite biosensor for the detection of *E. coli* in aqueous solutions based on enzyme amplification. The efficiency of the method was demonstrated in both solution and test strip format [40]. In this design, cationic AuNPs featuring quaternary amine head groups are electrostatically bound to an anionic enzyme, β-galactosidase, leading to inhibition of the enzyme activity. Upon binding of bacteria to the AuNPs, β-galactosidase is released restoring its activity. The binding event was quantified by colorimetric means. Using this strategy, bacteria at concentrations of 1 × 10^2^ bacteria/mL in solution and 1 × 10^4^ bacteria/mL in a field-friendly test strip format have been quantified (Figure 5) [40].

An immuno-chromatographic (IC) test strip against *E. coli* 0157 in enriched samples (raw beef, pork, bovine feces and swine feces) was developed by Jung *et al.* [107]. The test, fabricated in a sandwich format, utilizes a murine monoclonal antibody against *E. coli* O157:H7 conjugated to colloidal AuNPs. Sensitivity of the IC strip was assessed with a10 fold diluted *E. coli* O157:H7 sample with a range of 1.8 × 10^7^ to 1.8 colony-forming units (CFU)/mL in enriched raw beef. The detection limit was 1.8 × 10^5^ CFU/mL without enrichment and 1.8 CFU/mL after enrichment. 48 of pure bacteria cultures (32 *E. coli* strains and 16 non-*E. coli* strains) were tested to determine the specificity.

**Figure 6:**
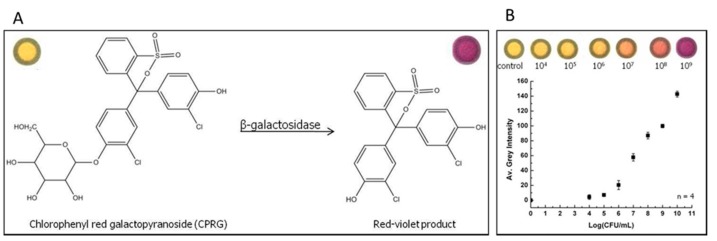
(**A**) Schematic shows the enzymatic reaction between galactosidase and chlorophenyl red galactopyranoside; (**B**) Calibration curve for detection of *E. coli* has been drawn for determination of the limit of detection for live bacterial assay (reprinted with permission from [99]; copyright 2015 American Chemical Society).

Three strains showed positive signal to *E. coli* O157:H7 by the IC strip while the other 29 *E. coli* serotypes were negative. Among the non-*E. coli* strains, only *Citrobacter amalonaticus* yielded a positive signal. The specificity of the strip was higher with pork samples (98.8%) than with bovine (87.9%) and swine (93.4%) feces samples. The assay results were in agreement with the traditional culture procedure for the analysis of samples enriched with *E. coli* O157:H7. In another work, Hossain *et al*. reported a paper test strip for detection of *E. coli* [101]. The strip was based on the measurement of intracellular enzymes (β-galactosidase or β-glucuronidase) activity. The test was fabricated on paper strips (0.5 × 8 cm), onto which either 5-bromo-4-chloro-3-indolyl-β-d-glucuronide sodium salt (XG), chlorophenol red β-galactopyranoside (CPRG) or both and FeCl_3_ were entrapped using sol– gel-derived silica inks in different zones via an ink-jet printing technique. The XG is broken down by β-glucuronidase into D-glucuronic acid and ClBrindoxyl followed by oxidation of the latter into ClBr-indigo dye, a blue product. On the other hand, CPRG (yellow colour) is broken down by β-galactosidase into chlorophenol red, a red-magenta product. The enzyme β-galactosidase has been widely used for counting total coliforms because the coliforms are generally β-galactosidase positive [108]. In this design [105] formation of red magenta on paper indicated the presence of coliforms. Most *E. coli* strains possess β-glucuronidase activity [109] but the pathogenic *E. coli* O157:H7 doesn’t [110]. Therefore, the formation of blue color on the paper strips indicated the presence of non-pathogenic *E. coli*, while absence of blue color (and presence of red-magenta) was used as an indicator for pathogenic *E. coli*. Using immunomagnetic NPs for selective pre-concentration, the limit of detection was 5 CFU/mL for *E. coli* O157:H7 and 20 CFU/mL for *E. coli* BL21, within 30 min without cell culturing. Jokerst *et al.* developed a paper-based sensor for colorimetric detection of foodborne pathogens by measuring the color change when an enzyme associated with the pathogen of interest reacts with a chromogenic substrate (Figure 6) [99]. When combined with an enrichment procedure step of 12 h or less, the paper-based device was capable of detecting bacteria at a concentration of 10^1^ CFU/cm^2^.

Several lateral flow strip test kits which enable low-cost detection of *E. coli* O157 in food are commercially available: MaxSignal^®^, RapidChek^®^, Gen-Probe^®^, IQuum^®^, Watersafe^®^, and others. The limit of detection of bacteria using such strips ranges from 10^4^ to 10^7^ CFU/mL without an enrichment step. The detection limit for *E. coli* O157:H7 is ~10^5^ CFU/mL. Yet, culturing steps of at least 8 h are mostly required in order to reach a detection limit of 1 CFU/mL [101].

**Figure 7:**
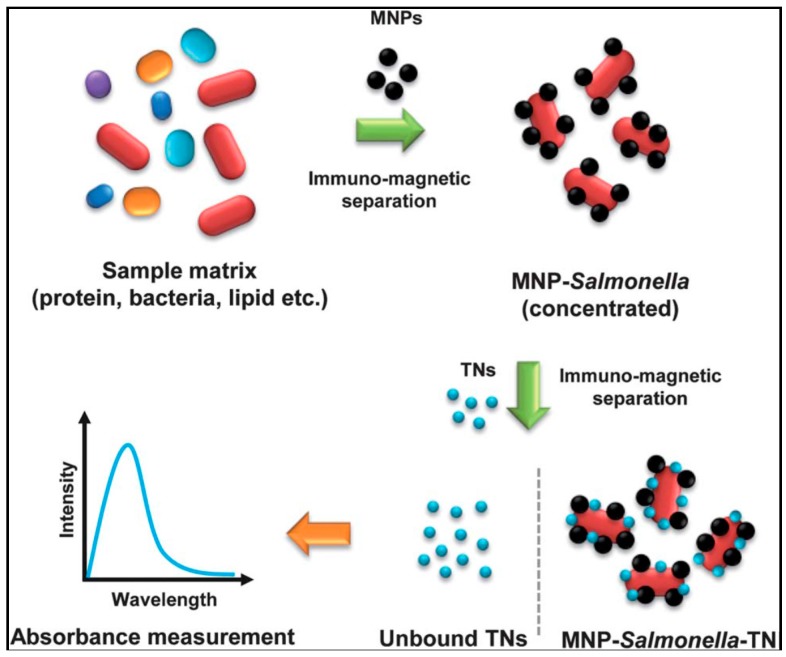
A schematic representation of the pathogenic bacteria detection method by using magnetic nanoparticles and optical nanoprobes (reproduced from [114] with permission of The Royal Society of Chemistry).

Another foodborne pathogen, *Salmonella* spp. causes one of the most common and widely distributed bacterial diseases, salmonellosis [111] with thousands of cases reported annually [111,112,113]. Most reported NP-based assays for *Salmonella* involve the use of immunomagnetic separation with immuno-modified magnetic NPs (MNPs). Joo et al detected *Salmonella* in milk by using MNPs and TiO_2_ nanocrystals [114]. In this design, *Salmonella* was selectively captured, concentrated and separated from solution by antibody-immobilized magnetic NPs (Figure 7). Subsequent binding of antibody-conjugated nanocrystals to the MNP–*Salmonella* complexes was monitored by absorbance measurement. The method enabled detection of 100 CFU/mL for *Salmonella* in milk.

Huang *et al.* demonstrated the use of amine-functionalized (AF) MNPs for rapid capture and removal of bacterial pathogens from water, food matrixes and urine samples [115]. The positive charges on the surface of AF-MNPs induce strong electrostatic interactions with the negatively charged sites on the surface of bacterial pathogens resulting in efficient adsorption of bacteria on the particle surface.

### 3.3. Detection of Pesticides

The increased use of pesticides in agriculture raises public concern regarding the safety of food products. Among pesticides, organophosphorus (OP) and carbamates (C) are the most widely employed, representing ~40% of the world market of this class of compounds [116,117]. Their mode of action involves inhibition of acetylcholinesterase (AChE) enzyme which catalyzes the hydrolysis of neurotransmitter acetylcholine [118,119]. Reference methods for these compounds include chromatographic techniques (GC and HPLC) and coupled chromatographic-spectrometric procedures such as GC-MS and HPLC-MS. These techniques are expensive, time-consuming, and are not easily adaptable for *in situ* monitoring. Screening of multiple samples for pesticide contamination at site locations requires sensitive, selective and robust methods that can be used on site [120]. To increase portability and improve detection capabilities several examples of NP-based assays have been developed as alternative to chromatographic techniques [121]. Despite many reports on sensors for pesticides, the application of these devices for the analysis of real food sample has been scarcely demonstrated.

**Figure 8:**
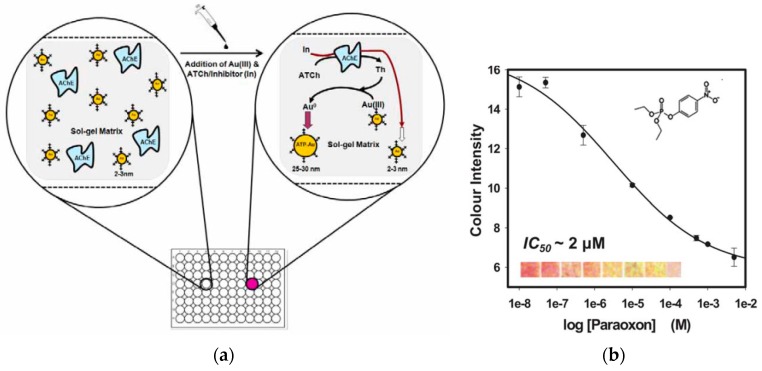
(**a**) Principle of a AChE colorimetric dipstick based on catalytic enlargement of AuNPs and (**b**) calibration curve for the detection of paraoxon (reproduced from [125] with permission of The Royal Society of Chemistry).

The most popular sensing configurations involve measurement of AChE inhibition with detection of the enzyme activity before and after exposure to pesticides. AChE activity is typically measured by the colorimetric Ellman assay [122]. Hossain *et al.* developed a paper-based solid phase sensor fabricated by inkjet printing the AChE enzyme within sol-gel derived silica layers onto paper [123]. Pesticides were detected by measuring the residual AChE activity on paper, by using the Ellman’s colorimetric assay. The detection was demonstrated on lateral flow and dipstick formats with detection limits of ~100 nM for paraoxon and 30 nM for aflatoxin B1, and a rapid response time (<5 min). In follow up work, Hossain *et al.* improved the design by depositing all the required reagents together with the enzyme onto paper [124]. Figure 8 shows an example of colorimetric “dipstick” bioassay based on AChE-catalyzed enlargement of AuNPs (3 nm) co-entrapped with the enzyme on paper [125]. Both the acetylthiocholine substrate and Au(III) salt were spotted on paper. Hydrolysis of the enzyme substrate generated thiocholine, which further reduced the Au(III) to AuNPs, inducing particle growth and resulting in an increase in color intensity. The color produced was correlated with enzyme inhibition by pesticides. A linear range from 500 nM to 1 mM was reported for paraoxon.

**Figure 9:**
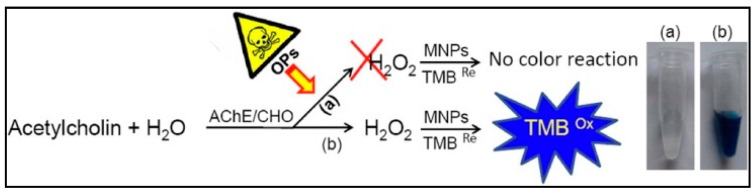
Sensing principle of the assay based on Fe_3_O_4_ magnetic nanoparticle for OPs detection (reprinted with permission from [126]; copyright 2015 American Chemical Society).

Liang *et al.* reported a colorimetric method using a bi-enzyme system, AChE and choline oxidase (ChO), and Fe_3_O_4_ MNPs with peroxidase mimetic activity to detect of OPs and nerve agents [126]. ChO catalyzed the conversion of the product of the AChE reaction, choline to hydrogen peroxide (H_2_O_2_). The produced H_2_O_2_ was then detected colorimetrically by a color change generated by the catalytic action of the MNPs on the oxidation of 3,5,3′,5′-tetramethylbenzidine (TMB). In the presence of pesticides, the enzymatic activity of AChE was inhibited and less H_2_O_2_ was produced. The decrease in the color intensity was used to quality AChE inhibition by pesticides (Figure 9).

**Figure 10:**
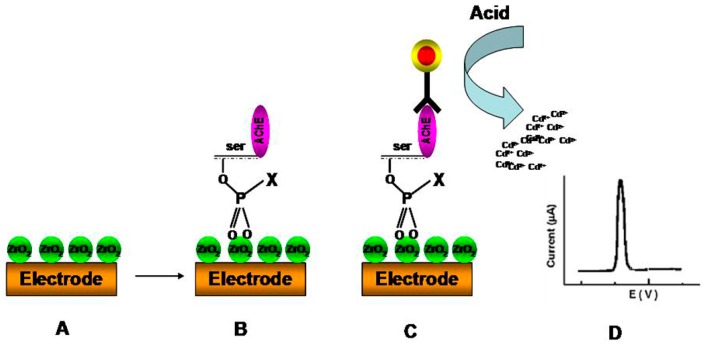
Electrochemical immunosensing of phosphorylated AChE using a ZrO_2_ NP-modified SPE (**A**) with selective capturing of phosphorylated AChE adducts (**B**) and detection via (**C**) Immunoreaction between bound phosphorylated AChE adducts and QD-labeled anti-AChE antibody (**D**) Representative voltammogram (reprinted with permission from [127]).

Detection of pesticides has also been realized with NP-based electrochemical immunosensing on low cost SPCE [127]. Figure 10 shows an example of a SPCE electrode functionalized with ZrO_2_ NPs for the detection of phosphorylated AChE. ZrO_2_NPs were used as sorbents for enzyme capture while quantum dots (QDs) were used as tags to label anti-AChE antibody and form a sandwich-like immunoreaction. The immunocaptured QD were determined by electrochemical stripping analysis of Cd ions after an acid-dissolution step of the QDs. The assay was used to detect AChE activity and paraoxon as an example of OP target and could be potentially extended to analysis in food.

**Figure 11:**
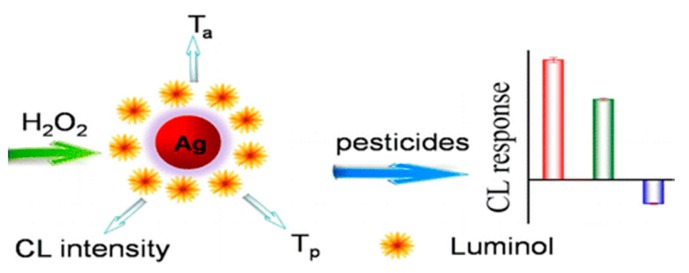
Chemiluminescence-based detection of pesticides using Lum-functionalized AgNPs (reprinted with permission from [128]; copyright 2015 American Chemical Society).

In addition to systems based on optical and electrochemical transduction, functionalized NPs can also be used to develop assays with chemiluminescent (CL) detection. A recent report demonstrates the use of luminol-functionalized AgNPs (Lum-AgNPs) as functional nanomaterials (Figure 11) for the detection of OP and C pesticides, including dimethoate, dipterex, carbaryl, chlorpyrifos, and carbofuran, at a concentration of 24 μg/mL [128]. The Lum-AgNPs were used in conjunction with a H_2_O_2_ based CL detection to generate a CL “fingerpring” related to each specific pesticide.

### 3.4. Detection of Metal Contaminants

Several metal ions such as arsenic, cadmium, lead and mercury are present into the environment and can be found as residues in food. Excess exposure to toxic metal ions can cause neurological, reproductive, cardiovascular, and developmental disorders [129]. Several NP-based sensors have been developed for detection of metal ions [129]. Most of these assays are based on Au and Ag NPs aggregation [34,42]. A portable lab-on-chip system for colorimetric detection of metal ions in water based on AuNP aggregation was developed by Zhao *et al.* [130]. The system provided detection limits of 30 ppb for Pb^2+^ and 89 ppb for Al^3+^. AuNPs based colorimetric detection was also demonstrated for Hg^2+^ [131,132,133,134,135], Cu^2+^ and Ag^+^ [136], Mn^2+^ [137], Cd^2+^ [138], Fe^3+^, Pb^2+^, Al^3+^, Cu^2+^, and Cr^3+^ [135]. Baxter *et al.* proposed a simple and economical process for fabricating gold electrodes by applying sintered AuNPs stabilised with 4-(dimethylamino)pyridine to filter paper and used this platform to detect Cu ions [93].

### 3.5. Detection of Mycotoxins

**Figure 12:**
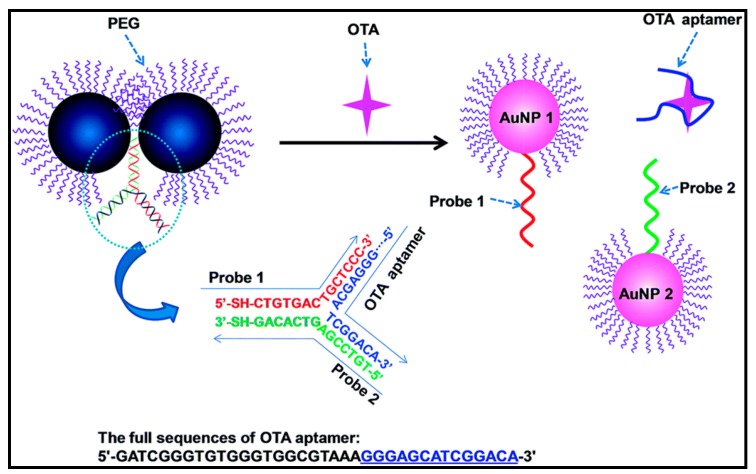
Sensing mechanism of the AuNP dimer-based colorimetric aptasensor for the detection of OTA (reproduced from [144] with permission of The Royal Society of Chemistry).

Toxin fungal metabolites known as mycotoxins can contaminate a wide range of agricultural commodities and are high priority targets for the development of new bioassays. It is estimated that at least 25% of the grain produced worldwide is contaminated with mycotoxins. Even small concentrations of mycotoxins can induce significant health problems including vomiting, kidney disease, liver disease, cancer and death [139]. Aflatoxins, ochratoxins, trichothecenes, zearalenone, fumonisins, tremorgenic toxins, and ergot alkaloids are examples of toxic mycotoxins. Mycotoxins have been implicated in development of cancer by the WHO-International Agency for Research on Cancer in 1993. Naturally occurring aflatoxins are classified as carcinogenic to humans (Group 1) whereas ochratoxins and fumonisins are classified as possible carcinogens (Group 2B) [140]. Aflatoxins are the most studied group of mycotoxins [140]. Another important toxin, ochratoxin A (OTA) is a type of mycotoxin which is produced by several species of *Aspergillus* and *Penicillium* fungi and can be found in a wide variety of food matrices such as cereals, dried fruits, coffee, cocoa, spices, beer, wine and grape juice [141]. Hosseini *et al.* developed a AuNP-based aptasensor for detection of aflatoxin B1. The sensor measured AuNP aggregation due to desorption of the aflatoxin B1 aptamer from the surface of AuNPs after the aptamer-target interaction resulting in the color change of AuNPs from red to purple. A detection limit of 7 nM with a linear range from 80 to 270 nM was reported [142]. A similar approach was described by Luan *et al.* for the detection of aflatoxin B2 [143]. In the absence of aflatoxin B2, the random coil structure of the aptamer stabilizes the surface of AuNPs, which shows a red color under high NaCl conditions. In presence of aflatoxin B2, formation of aflatoxin B2-aptamer conjugate destabilizes the AuNPs from NaCl-induced aggregation, changing the color of the dispersion. The method was characterized by a linear dynamic range from 0.025 to 10 ng·mL^−1^, and a detection limit of 25 pg·mL^−1^. Xiao *et al.* demonstrated a colorimetric detection method based on disassembly of AuNP dimers for the detection of OTA (Figure 12). This system was characterized by a low detection of 0.05 nM, with a dynamic range from 0.2 to 250 nM [144]. The proposed sensor was applied for detection of OTA in red wine. The OTA concentration determined by this method was consistent with the result obtained with a commercially available ELISA kit. Soh *et al.* described a colorimetric method for detection of OTA by using aptamer controlled growth of AuNPs [145]. In this system the aptamer-target interactions control the amount of aptamer strands adsorbed on the surface of AuNPs. Depending on the surface coverage, AuNPs grow into morphologically varied nanostructures resulting in different colored solutions. AuNPs with low aptamer coverage produced red-colored solutions, whereas AuNPs with high aptamer coverage produced blue colored solutions. The detection limit for OTA using this method was 1 nM.

**Figure 13:**
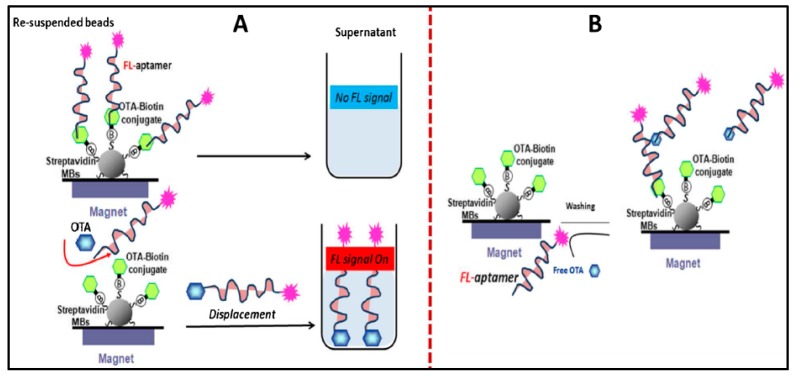
Schematic represents the principle of the assay for the fluorescence detection methodologies. (**A**) displacement assay; (**B**) competition assay. (With permission from Springer Science and Business Media: Analytical and Bioanalytical Chemistry. Development of an aptasensor based on a fluorescent particles-modified aptamer for ochratoxin A detection. 407, 2015, 7815–7822 Hayat, A.; Mishra, R.K.; Catanante, G.; Marty, J.L. Scheme 1.).

Other assays are using MNPs [146,147] to design immunomagnetic separation and detection platforms for mycotoxins. Electrochemical immunosensors and aptasensors employing magnetic NPs as immobilization support have been designed for the detection of mycotoxins such as OTA and aflatoxin [75,148]. Figure 13 shows an example of aptasensor platform for detection of OTA. A generic fluorescent aptasensing platform was designed by employing carboxy-modified fluorescent particles as a signal generating probe and magnetic particles as a solid separation support [149]. Table 1 provides the summary of analytical characteristics of portable NP-based sensors reported in literature for the detection of food contaminants.

**Table 1:** Summary of analytical characteristics of reviewed studies on portable NP-based sensors for the detection of food contaminants.

	Analyte	Assay Format	NPs Used	Detection Principle	Detection Limit	Dynamic Range	Refs.
**Microbial Contaminants**	*E. coli* (XL1)	Colorimetric	AuNPs	Binding of bacteria to AuNPs leads the release of the enzyme attached on particles, restoring its activity	1 × 10^2^ bacteria/mL	1 × 10^2^–1 × 10^7^ bacteria/mL	[40]
	*E. coli* O157:H7	Colorimetric	MNPs	The activity of intracellular enzymes measured	5 cfu/mL	-	[101]
	*E. coli* BL 21				20 cfu/mL		
	*E. coli* O157:H7	Colorimetric	AuNPs	Monoclonal antibody is conjugated to the colloidal AuNPs, and the sandwich format is utilized	1.8 cfu/mL	1.8 cfu–1.8 × 10^7^ cfu/mL	[107]
	*Salmonella*	Colorimetric	MNPs and TiO_2_ nanocrystals	Salmonella were captured by antibody-immobilized magnetic nanoparticles and separated from solution, subsequent binding of antibody-conjugated TNs to the MNP–Salmonella complexes increased absorption	>100 cfu/mL	10^8^–10^2^ cfu/mL	[114]
	*E. coli*	Colorimetric	MNPs	Amine-functionalized (AF) MNPs used for rapid capture and removal of bacterial pathogens by using plate counting method	-	-	[115]
	*Sarcina lutea*						
	*Proteus vulgaris*						
**Pesticides**	Paraoxon	Colorimetric	AuNPs	Bioassay based on AChE-catalysed enlargement of AuNPs co-entrapped with the enzyme on paper. Hydrolysis of the enzyme substrate generated thiocholine, which further reduced the Au(III) to AuNPs, inducing particle growth and resulting in an increase in color intensity	0.5 μM	500 nM–1 mM	[125]
	Sarin	Colorimetric	MNPs	ChO catalyzed the conversion of the product of the AChE reaction to hydrogen peroxide (H_2_O_2_). Produced H_2_O_2_ was then detected by the catalytic action of the MNPs on the oxidation of its substrate generating a color change.	1 nM	-	[126]
	Methyl-paraoxon				10 nM		
	Acephate				5 μM		
	Phosphorylated AChE	Electrochemical	ZrO_2_ NPs	ZrO_2_NPs were used as sorbents for enzyme capture while quantum dots (QDs) were used as tags to label anti-AChE antibody and form a sandwich-like immunoreaction. The immunocaptured QD were determined by electrochemical stripping analysis of Cd ions after an acid-dissolution step of the QDs	8.0 pM	10 pM to 4 nM	[127]
	Dimethoate	Chemiluminescent	AgNPs	The array is based on the triple-channel properties of luminol-functionalized AgNPs and hydrogen peroxide chemiluminescent (CL) system containing CL intensity	24 μg/mL	-	[128]
	Dipterex						
	Carbaryl						
	Chlorpyrifos						
	Carbofuran						
**Metals**	Pb^2+^	Colorimetric	AuNPs	Analyte induced aggregation/disaggregation phenomena of AuNPs	400 μM	-	[35]
	Hg^2+^	Colorimetric	AuNPs	Analyte induced aggregation/disaggregation phenomena of AuNPs	0.8 nM	50–250 nM	[131]
	Hg^2+^	Colorimetric	AuNPs	Analyte induced aggregation/disaggregation phenomena of AuNPs	8 nM	0.01–5 μM	[133]
	Hg^2+^	Colorimetric	AuNPs	Analyte induced aggregation/disaggregation phenomena of AuNPs	50 nM	25–750 nM	[134]
	Hg^2+^	Colorimetric	AuNPs	Analyte induced aggregation/disaggregation phenomena of AuNPs	53 nM	33–300 nM	[137]
	Pb^2+^				1 6 nM	16 × 10^−9^ to 100 × 10^−9^ M	
	Hg^2+^		AgNPs		16 nM	16–660 nM	
	Mn^2+^					16 × 10^−9^–50 × 10^−8^ M	
	Cd^2+^	Colorimetric	AuNPs	Analyte induced aggregation/disaggregation phenomena of AuNPs	16.6 nM	0.5–16 μM	[138]
**Mycotoxins**	Aflatoxin B1	Colorimetric and Chemiluminescence	AuNPs	Analyte induced aggregation/disaggregation phenomena of AuNPs	7 nM	80–270 nM	[142]
	Aflatoxin B2	Colorimetric	AuNPs	Analyte induced aggregation/disaggregation phenomena of AuNPs	25 pg/mL	0.025–10 ng/mL	[143]
	Ochratoxin A	Colorimetric	AuNPs	Analyte induced aggregation/disaggregation phenomena of AuNPs	0.05 nM	0.2 nM to 250 nM	[144]
	Aflatoxin B1 (AFB)	Colorimetric	AuNPs	Competitive immunoassay between AFB modified magnetic beads and free AFB for AuNPs labelled antibodies.	12 ng/L	20–800 ng/L	[146]
	Ochratoxin A (OTA)	Fluorescent	Fluorescent particles	OTA-MBs (magnetic beads) were immobilized inside the wells and the analysis was performed by adding the fluorescent particles-modified aptamer which competed with the immobilized OTA and OTA in solution. The presence of OTA in solution prevented binding of the immobilized OTA to the aptamer, leading to a decrease of the fluorescence signal.	0.005 nM	0.1–150 nM	[149]

## 4. Conclusions and Future Directions

NPs and nanostructures have demonstrated ability to significantly enhance detection capabilities of analytical devices. A variety of platforms based on these materials have been reported, and many have shown high sensitivity and low detection limits. The design features indicate that these can be potentially used as portable instrumentation. However, most capabilities have been demonstrated with synthetic samples in laboratory conditions. Some detection protocols involve the use of sensitive reagents and multiple-step procedures which increase measurement time and cost and make field implementation difficult. Translation of this technology into the food safety and regulatory field requires rigorous validation with conventional methodologies, testing of real samples and careful evaluation of interferences. The effect of environmental parameters, storage and operational stability in field conditions should also be established. Moreover, concerns have been raised regarding the potential toxicity of nanomaterials. This aspect should be further considered before these platforms can be introduced into the marketplace.

Analysis of food is a difficult problem due to the inherent complexity of these samples. Challenges for implementation of emerging technologies as viable platforms for assessing food safety and quality are related to interferences and the need for sample preparation. Most developed sensors require sample preparation steps. Some advances have been made with the use of MNPs for immunoseparation. In the future, the integration of sample extraction and separation units with the sensing platforms would greatly improve portability for field use. The long term storage is another challenge especially for systems that include biological sensing components such as enzymes and antibodies. Achieving high specificity is also critical to minimize background signals and reduce the likelihood of false-positive results. Miniaturization, automation, multidetection capabilities and an effort to lower the cost per assay are some of the current trends in this field. The use of inexpensive materials such as paper to build these sensors has demonstrated potential as field-portable devices but their functionality for the analysis of complex food samples and the need for sample pretreatment are yet to be demonstrated. The performance of disposable SPC electrodes has been improved with the use of nanomaterials. Validation and testing of statistically-relevant sample numbers, comparability and inter-laboratory studies to demonstrate robustness of such platforms are the next critical steps for achieving industry acceptance and regulatory approvals. Future work to adapt these sensors so that they can be attached to food packaging to indicate contamination would be highly valuable for on-line control and safety of processed and stored food. Increased portability can also be achieved through connectivity and integration with largely used communication devices such as cell-phones and tablets. However, development of cybersensors for food monitoring is still in infancy and constitutes a fertile area for future investigation.
